# Neurocognitive functioning and emotional recognition in first-episode psychosis: protocol for a follow- up study

**DOI:** 10.1192/j.eurpsy.2024.1501

**Published:** 2024-08-27

**Authors:** D. Bošnjak Kuharić, P. Makaric, K. Brozic, L. Tomasic, A. Savic, D. Ostojic, M. Rojnic Kuzman

**Affiliations:** ^1^Department for psychotic disorders- female; ^2^Department for affective disorders; ^3^Department for first episode psychosis, University Psychiatric Hospital Vrapce; ^4^Department of Psychiatry and Psychological Medicine, Zagreb University Hospital Centre, Zagreb, Croatia

## Abstract

**Introduction:**

Although deficits in neurocognitive functioning and emotional recognition impact treatment outcomes in schizophrenia since the development of the first psychotic episode (FEP), there is still a lack of longer follow-up studies showing the course of these deficits over time.

**Objectives:**

Our objective is to investigate the changes of cognitive functioning over years in a cohort of patients, since their FEP.

**Methods:**

This study is developed as a follow-up of the project Biomarkers in schizophrenia- integration of complementary methods in longitudinal follow-up of FEP, that was conducted in several Croatian psychiatric clinics during the period from 2014 to 2019. A cohort of patients with FEP took part in the project with psychopathology, neurocognitive functioning and emotional recognition assessment at two time points- at baseline, during the subacute phase of a psychotic episode, and after 18 months of follow-up. In this study, patients with FEP who completed the baseline assessment of the project (n=159), will be contacted and included in the follow- up. Follow-up assessment will consist of sociodemographic data including information of their treatment so far, battery of neurocognitive tests (Rey Auditory Verbal Learning Test, Rey–Osterrieth complex figure Test, Wechsler paired memory, trail making test a & b, Digit symbol, Digit span, Semantic & Phonetic Fluency, Stroop 1, 2, 3 and Block design test), emotional recognition test (Penn Emotion Recognition Task) and several scales assessing psychopathology (Positive and Negative Syndrome Scale, Self-evaluation of Negative Symptoms, Scale for the Assessment of Negative Symptoms), functioning (Global Assessment of Functioning, WHO Disability Assessment Scale 2.0), quality of life and recovery. The study is funded by the University of Zagreb programmes (Project No. 10106-23-2394).

**Results:**

We plan to analyze the differences between the three time points (baseline, 18 months, 5 years), taking in account possible correlations with psychopathology, functioning, quality of life and different treatment options.

**Conclusions:**

Identifying specific deficits can help in providing more effective treatment plan including various interventions that can improve treatment outcomes in schizophrenia.

**Disclosure of Interest:**

None Declared

